# Description of the Human Atrial Action Potential Derived From a Single, Congruent Data Source: Novel Computational Models for Integrated Experimental-Numerical Study of Atrial Arrhythmia Mechanisms

**DOI:** 10.3389/fphys.2018.01211

**Published:** 2018-09-07

**Authors:** Michael A. Colman, Priyanka Saxena, Sarah Kettlewell, Antony J. Workman

**Affiliations:** ^1^Leeds Computational Physiology Lab, School of Biomedical Sciences, University of Leeds, Leeds, United Kingdom; ^2^Institute of Cardiovascular and Medical Sciences, College of Medical, Veterinary and Life Sciences, University of Glasgow, Glasgow, United Kingdom

**Keywords:** atrial fibrillation, human atrial model, ion channel currents, computational model, action potential

## Abstract

**Introduction:** The development of improved diagnosis, management, and treatment strategies for human atrial fibrillation (AF) is a significant and important challenge in order to improve quality of life for millions and reduce the substantial social-economic costs of the condition. As a complex condition demonstrating high variability and relation to other cardiac conditions, the study of AF requires approaches from multiple disciplines including single-cell experimental electrophysiology and computational modeling. Models of human atrial cells are less well parameterized than those of the human ventricle or other mammal species, largely due to the inherent challenges in patch clamping human atrial cells. Such challenges include, frequently, unphysiologically depolarized resting potentials and thus injection of a compensatory hyperpolarizing current, as well as detecting certain ion currents which may be disrupted by the cell isolation process. The aim of this study was to develop a laboratory specific model of human atrial electrophysiology which reproduces exactly the conditions of isolated-cell experiments, including testing of multiple experimental interventions.

**Methods:** Formulations for the primary ion currents characterized by isolated-cell experiments in the Workman laboratory were fit directly to voltage-clamp data; the fast sodium-current was parameterized based on experiments relating resting membrane potential to maximal action potential upstroke velocity; compensatory hyperpolarizing current was included as a constant applied current. These formulations were integrated with three independent human atrial cell models to provide a family of novel models. Extrapolated intact-cell models were developed through removal of the hyperpolarizing current and introduction of terminal repolarization potassium currents.

**Results:** The isolated-cell models quantitatively reproduced experimentally measured properties of excitation in both control and pharmacological and dynamic-clamp interventions. Comparison of isolated and intact-cell models highlighted the importance of reproducing this cellular environment when comparing experimental and simulation data.

**Conclusion:** We have developed a laboratory specific model of the human atrial cell which directly reproduces the experimental isolated-cell conditions and captures human atrial excitation properties. The model may be particularly useful for directly relating model to experiment, and offers a complementary tool to the available set of human atrial cell models with specific advantages resulting from the congruent input data source.

## Introduction

The incidence of atrial fibrillation (AF) is growing in epidemic proportions (Ceornodolea et al., 2017): the current prevalence of 2% in western countries is more than double that of over a decade ago (Zoni-Berisso et al., 2014) and its association with aging has led to projections of significant increase in the next few decades (Krijthe et al., 2013). AF increases the risk of death, mainly as a consequence of the associated four to fivefold increased risk of stroke, and results in significant costs to worldwide healthcare systems (Casajuana et al., 2018). Presently available treatment strategies are far from ideal: pharmacological intervention has sub-optimal efficacy in patients, and also risks adverse effects in certain patient groups; catheter ablation is invasive and may require repeated procedures (Kirchhof et al., 2016; Kirchhof and Calkins, 2017). The development of improved diagnosis, management, and treatment strategies for AF is therefore a significant and important challenge in order to improve quality of life for millions and reduce the substantial socio-economic costs of the condition.

The mechanisms driving the sustained rapid and irregular excitation during AF are controlled partly at the cellular level by the non-linear interactions of multiple ion currents underlying the action potential (AP), and thus these currents also influence the impact of pharmacological or surgical interventions. It is therefore vital to fully characterize the dynamics of these ion currents. Compounding the problem, expression and kinetics of these currents can be highly variable (e.g., Lawson et al., 2018), are modified by autonomic control (e.g., Chen and Tan, 2007; Workman, 2010), and can remodel in the long-term presence of AF (e.g., Workman et al., 2001); excitation dynamics at the tissue-scale may also be highly non-linear and unpredictable from single-cell studies alone (e.g., Colman et al., 2017b). It is therefore a significant challenge to comprehensively quantify the role of ion currents (and their variation, modulation, and remodeling) in the mechanisms of atrial arrhythmia.

Computational modeling has proved to be an increasingly valuable tool to assess and dissect the impact of ion channel function and anatomical structure on normal and arrhythmic human atrial electrical dynamics (Colman et al., 2014; Trayanova, 2014; Heijman et al., 2016): the number of simulation studies has significantly increased over the last decade and there are currently multiple contemporary models of the human atrial AP and calcium handling available (e.g., Courtemanche et al., 1998; Nygren et al., 1998; Maleckar et al., 2009; Grandi et al., 2011; Koivumäki et al., 2011; Colman et al., 2013). Despite the successes of simulation studies, human atrial cell models are in general less well parameterized than ventricular cell models, and they still rely on model components formulated on data from different sources, cell-types, and even species (Wilhelms et al., 2013); integration of components from different sources is non-trivial and often requires enforced parameter modification. Even integration of human atrial specific data from multiple sources is non-trivial due to the challenges of obtaining reliable data (Nattel, 2006), including substantial inter-patient, inter-isolate, and inter-cell variability. Also, subtle inter-laboratory differences, as well as variations in operative techniques and concomitant therapies, may influence the outcome of experiments in major ways, making multi-center studies challenging and providing uncertainty in the combination of mathematical descriptions based on data from multiple laboratories. Moreover, the recent trend has been toward introducing more components and additional complexity and sophistication; whereas this has proved useful in teasing apart ionic contributions to health and disease, it nevertheless provides more potential sources of error and reliance on a larger set of unknowns and/or *ad hoc* parameters. There is therefore motivation to derive a computational model based on human atrial data with the fewest non-specific additional components, and also specifically on data from a single source (i.e., consistent cell source and experimental environment and protocols).

There are numerous further challenges in obtaining reliable experimental data for developing a human atrial-specific cell model. These include inherent difficulties in acquiring human atrial tissues and acutely isolating cardiomyocytes from them, since they cannot be arterially perfused with enzymes. The “chunk technique” (Yue et al., 1996), in which small chunks of tissue are superfused with enzymes, is used to overcome this problem, but also has recognized limitations including potential disruption of ion currents such as the delayed rectifier potassium currents *I*_Kr_ and/or *I*_Ks_ (Yue et al., 1996; Workman et al., 2006), relatively low cell yield, and often an unphysiologically depolarized resting membrane potential (RMP; Escande et al., 1987; Bénardeau et al., 1996; Van Wagoner et al., 1999; Dobrev et al., 2001; Workman et al., 2006) to the extent that the fast sodium current (*I*_Na_) may be unavailable. RMP depolarization can be mitigated in various ways, none without limitations, including injecting a constant background (hyperpolarizing) current during recording, as used routinely in the Workman laboratory (WL). This method permits full repolarization of the AP and thus *I*_Na_ availability, but must be used sparingly and with caution; see Workman et al. (2006) for details. Computational models of atrial cells therefore do not necessarily directly translate to the isolated-cell experiments on which they are validated and which they themselves inform. There is therefore also motivation to develop a cell model which reproduces specifically the conditions of isolated whole-cell-patch current- and voltage-clamp experiments, for full congruence between experimental and simulation studies.

The aim of this study was to develop a human atrial isolated- and intact-cell model based primarily on specific human atrial cellular data from the WL, with motivation to introduce the fewest additional components from other sources. The model was tested for its ability to reproduce experimental observations for AP properties and their modulation by pharmacological and dynamic-clamp interventions. Furthermore, the descriptions of the set of the primary currents were integrated with contemporary human atrial cell models to provide a novel set of modified cell models, and used to assess the importance of the isolated-cell environment in AP morphology and response to various interventions. The resulting models therefore form a complementary tool to the available set of human atrial cell models, providing both a minimal approach with the emphasis on congruent input data which allows direct interaction between simulation and experiment, and a family of cell models suitable for mechanistic evaluation of atrial arrhythmias.

## Materials and Methods

In this study, novel formulations of the primary ion currents underlying human atrial electrophysiology and characterized in the WL were developed (sections “Isolated-Cell Experiments” and “Ion Current Formulations”). The formulations were integrated with multiple contemporary human atrial cell models, including both isolated- and intact-cell variants (section “Computational models”). The Courtemanche et al. (1998) model (hAM_CRN), Nygren et al. (1998) model (Nygren-Giles, hAM_NG), and Grandi et al. (2011) [Grandi-Bers, hAM_GB – specifically, the implementation of Chang et al. (2014)] were selected as these represent the baseline cell models underlying the primary distinct lines of human atrial cell model development [not including spatial cell models such as Koivumäki et al. (2011) and Voigt et al. (2014)]. Full model equations and parameters are provided in the **Supplementary Material** and model code in C/C++ is available from the GitHub repository^1^.

### Isolated-Cell Experiments

Isolated-cell experiments described pertain to both previously published and unpublished data characterizing the primary human atrial ion currents: fast sodium current (*I*_Na_); transient-outward current (*I*_to_); L-type calcium current (*I*_CaL_); ultra-rapid, or sustained, potassium current (*I*_Kur/sus_); and the time-independent potassium current (*I*_K1_); as well as additional intracellular calcium concentration ([Ca^2+^]_i_) data associated with the calcium transient (CaT).

A summary of the experimental conditions associated with each study is provided in **Table 1** and described briefly. Right atrial tissues were obtained from consenting patients undergoing cardiac surgery between 1999 and 2018. All patients were in sinus rhythm unless otherwise stated, and were taking a variety of drug treatments as detailed in the following publications. The whole-cell patch clamp technique and fluorescence microscopy were used to record ion currents, APs, effective refractory period (ERP), and intracellular Ca^2+^ concentration ([Ca^2+^]_i_), at 35–37°C, in the absence and presence of a variety of drugs, from atrial myocytes enzymatically isolated from these tissues. Procedures were approved by the West of Scotland Research Ethics Service (REC 99MC002 and REC 17/WS/0134). The chunk method of cell isolation used is detailed in Workman et al. (2001), and all the experimental conditions, patch-clamp configurations, recording protocols, and solutions used, are included in Workman et al. (2006, 2012) and Marshall et al. (2012). Specific data on individual ion currents, APs, or ERP are derived from the following WL published sources: APs, current magnitudes, and ERP: Workman et al. (2001, 2006) (referred to in short as WL_2001; WL_2006); *I*_K1_: Marshall et al. (2012) (referred to as WL_2012_IK1); *I*_to_ and *I*_sus_: Workman et al. (2012) (WL_2012_ITO); *I*_CaL_: Pau et al. (2007) (WL_2007_ICAL). WL_2006 was considered the most representative collective data and was therefore used to set the relative magnitudes of the currents in the computational models.

**Table 1:** Control experimental conditions used for human atrial cell electrophysiological recordings, including intra- and extra-cellular ionic constituents, [Ca^2+^]_i_-buffering and temperature (all closely matching and within mammalian physiological ranges), recording configurations and measurements taken.

Study	WL_2001 (Workman et al., 2001)	WL_2006 (Workman et al., 2006)	WL_2007 _ICAL (Pau et al., 2007)	WL_2012 _IK1 (Marshall et al., 2012)	WL_2012 _ITO (Workman et al., 2012)
Intracellular solution constituents (mM, pH)	L-aspartate (110), KCl (20), MgCl_2_ (1.0), EGTA (0.15), Na_2_ATP (4.0), Na_2_GTP (0.4), HEPES (5.0), pH 7.3	For ruptured-patch: K-aspartate (110), KCl (20), MgCl_2_ (1.0), EGTA (0.15), Na_2_ATP (4.0), Na_2_GTP (0.4), HEPES (5.0), nystatin (0.18); pH 7.3 For perforated-patch: KCl (30), HEPES (5.0), MgCl_2_ (1.0), K methane-sulfonic acid (100), NaCl (5.0); pH 7.3	KCl (30), HEPES (5), MgCl_2_ (1), K methane-sulfonic acid (100), NaCl (5), nystatin (0.18); pH 7.3	K-aspartate (110), KCl (20), MgCl_2_ (1.0), EGTA (0.15), Na_2_ATP (4.0), Na_2_GTP (0.4), HEPES (5.0); pH 7.3	K-aspartate (130), KCl (15), NaCl (10), MgCl_2_ (1), HEPES (10), EGTA (0.1); pH 7.3

Extracellular solution constituents (mM, pH)	NaCl (130), KCl (4.0), CaCl_2_ (2.0), MgCl_2_ (1.0), glucose (10), HEPES (10), pH 7.4	NaCl (130), KCl (4.0), CaCl_2_ (2.0), MgCl_2_ (1.0), glucose (10), HEPES (10); pH 7.4	NaCl (130), KCl (4.0), CaCl_2_ (2.0), MgCl_2_ (1.0), glucose (10), HEPES (10), pH 7.4	NaCl (130), KCl (4.0), MgCl_2_ (1.0), CaCl_2_ (2.0), glucose (10), HEPES (10); pH 7.4	NaCl (140), KCl (4.0), CaCl_2_ (1.8), MgCl_2_ (1.0), glucose (11), HEPES (10); pH 7.4

LLJP correction	Yes	Yes	Yes	Yes	Yes

Recording temperature	35–37°C	35–37°C	35–37°C	35–37°C	35–37°C

[Ca^2+^]_i_-buffering	Minimal (0.15 mM EGTA)	Minimal (0.15 mM EGTA for ruptured-patch; zero EGTA for perforated-patch)	Minimal (zero EGTA as perforated-patch)	Minimal (0.15 mM EGTA)	Minimal (0.10 mM EGTA)

Cell isolation enzyme	Collagenase (Type 1, Worthington, 400 U/ml)	Collagenase (Type 1, Worthington, 400 U/ml)	Collagenase (Type 1, Worthington, 400 U/ml)	Collagenase (Type 1, Worthington, 400 U/ml)	Collagenase (Type 1, Worthington, 400 U/ml)

Patch configuration	Ruptured patch	Ruptured patch Nystatin-perforated patch	Nystatin-perforated patch	Ruptured patch	Ruptured patch

Patch-clamp amplifier	Axopatch-1D (Axon)	Axopatch-1D (Axon)	Axopatch-1D (Axon)	Axopatch-1D (Axon)	AxoClamp 2B (Axon)

Recording configuration	Current-clamp Voltage-clamp	Current-clamp Voltage-clamp	Current-clamp Voltage-clamp	Voltage-clamp	Current-clamp Voltage-clamp Dynamic-clamp

Measurements	APs, ERP, *I*_CaL_, *I*_TO_, *I*_K1_, *I*_KSUS_	APs, ERP, *I*_CaL_, *I*_TO_, *I*_K1_, *I*_KSUS_	APs, ERP, *I*_CaL_	*I*_TO_, *I*_K1_, *I*_KSUS_	APs, *I*_TO_


*LLJP, liquid-liquid junction potential; AP, action potential; ERP, cellular effective refractory period; *I*_CaL_, L-type Ca^2+^ current; *I*_to_, transient outward K^+^ current; *I*_K1_, inward rectifier K^+^ current; *I*_sus_, sustained outward K^+^ current (mainly *I*_Kur_).*

*I*_Na_ data were derived using recent unpublished data obtained from human atrial isolated myocytes (by Priyanka Saxena in the WL) on the relationship between current-clamped RMP and AP phase 0 maximum upstroke velocity (*dV*/*dt*_max_) as a surrogate for the voltage-dependence of *I*_Na_ (which cannot be directly measured accurately) in these cells. [Ca^2+^]_i_ data (unpublished) were recently recorded in human atrial isolated myocytes (by Sarah Kettlewell in the WL), using identical techniques to those employed earlier in rabbit atrial myocytes in this laboratory (Kettlewell et al., 2013).

Only specific data on the ion currents were used to derive the mathematical model; AP, excitation, and intervention data were used for validation and comparison only, and not to further train the model.

### Ion Current Formulations

#### Workman-Laboratory Characterized Currents: *I*_K1_ and *I*_hyp_

**FIGURE 1:**
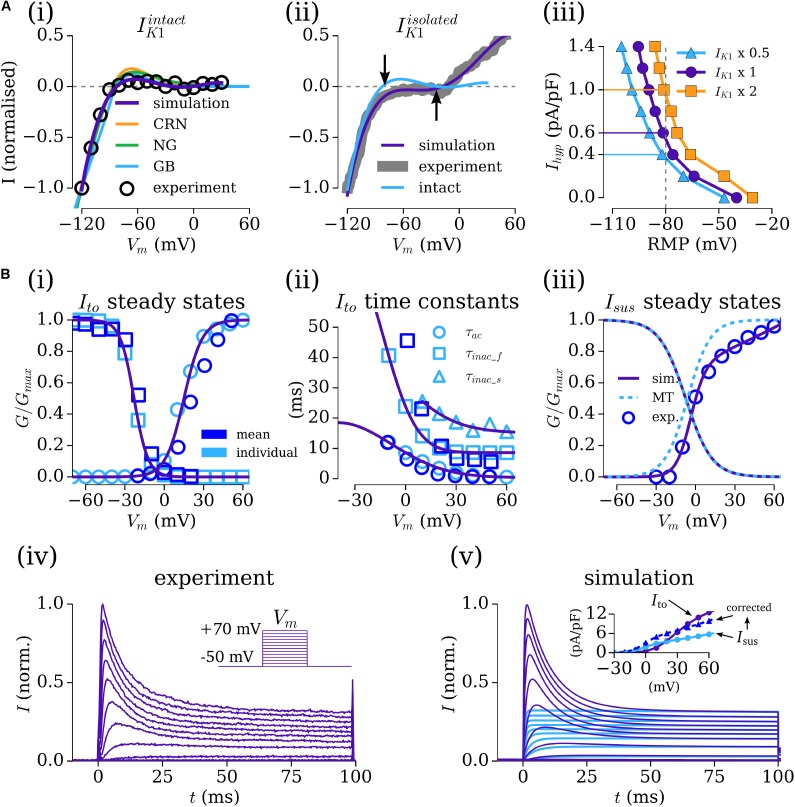
Fitting the Workman-lab K^+^ characterized currents. **(A)** Fitting *I*_K1_ and the hyperpolarizing current (*I*_hyp_). **(i)** *I*_K1_ measured in the WL_2012_IK1 study (Marshall et al., 2012), considered “intact” (black circles) and simulation fits from the new formulations (purple) and the three human atrial models [orange – CRN(Courtemanche et al., 1998); green – NG (Nygren et al., 1998); blue – GB (Grandi et al., 2011)]. **(ii)** *I*_K1_ measured in the WL_2006 (Workman et al., 2006) study, considered “isolated” (gray) and model fit (purple). The form of the “intact” current is shown for reference, with arrows indicating the difference in reversal between the intact and isolated variants. **(iii)** Relationship between resting membrane Potential (RMP) and *I*_hyp_ at three different amplitudes of *I*_K1_^isolated^ (baseline – closed circles, purple; *I*_K1_ × 2 – closed squares, orange; *I*_K1_ × 0.5 – closed triangles, blue). **(B,i–iii)** Fitting of the gating variable parameters for *I*_to_ and *I*_sus_ to the voltage-clamp data of WL_2012_ITO (Workman et al., 2012) **(iv)** and resulting simulated current traces **(v)** and IV relationship (inset). Open, light blue symbols refer to values calculated from fitting to individual current traces. Open, dark blue symbols refer to mean current data from WL_2012_ITO (Workman et al., 2012). The formulation from Maleckar et al. (2009) is shown for reference in panel **iii**. In the inset for panel **iv**, *I*_to_ is shown in purple, *I*_sus_ in light blue, and corrected *I*_sus_ in dark blue. Figure created using data from: Workman et al. (2006, 2012) and Marshall et al. (2012).

The current–voltage (IV) relationship for the time-independent current, *I*_K1_, was considered from both WL_2012_IK1 [**Figure 1A(i)**] and WL_2006 [**Figure 1A(ii)**], where the former data correspond to Ba^2+^-sensitive specific *I*_K1_, and the latter may more accurately refer to a general voltage-dependent, time-independent current. Integrating the formulation of the current from WL_2012_IK1 with the background currents of the selected baseline human atrial cell models led to maintained RMP of below -70 mV in all models. The apparent reversal in the WL_2006 data and formulation of approximately -40 mV [**Figure 1A(ii)**] was necessary to result in the unphysiologically depolarized RMP of isolated-cells. These formulations were therefore suitable for use in the intact- and isolated-cell models, respectively, and are thus referred as *I*_K1_^intact^ (WL_2012_IK1 data) and *I*_K1_^isolated^ (WL_2006 data) for clarity. The IV relationship associated with *I*_K1_ in the primary, independent human atrial cell models is shown for reference in the *I*_K1_^intact^ panel [**Figure 1A(i)**].

The relationship between RMP and the magnitude of the hyperpolarizing current (*I*_hyp_) for isolated-cell models with three different magnitudes of *I*_K1_ (0.5, 1, and 2 × maximum of -4 pA/pF at -120 mV; Workman et al., 2006) demonstrates hyperpolarizing currents of 0.4–1.4 pA/pF were necessary for maintaining a RMP of ∼-80 mV [**Figure 1A(iii)**], congruent with the magnitude of current used and reported in the isolated-cell experiments. An *I*_hyp_ of 0.63 pA/pF was used with the default scaling of *I*_K1_ for the baseline cell model, giving a RMP of -82 mV when integrated with the hAM_CRN calcium handling system and background currents. This value of *I*_hyp_ closely matched that reported in experiments, e.g., 0.62 pA/pF (Workman et al., 2006). This value also maintained a RMP of ∼-80 mV when combined with the background currents and calcium handling system in the hAM_NG (-75 mV) and hAM_GB (-80 mV) models. To ensure consistent conditions between models, this value of *I*_hyp_ was maintained rather than imposing identical RMP.

#### Workman-Laboratory Characterized Currents: *I*_to_ and *I*_sus_

The formulations for the transient-outward (*I*_to_) and sustained/ultra-rapid potassium (*I*_sus_/*I*_Kur_) currents were derived from the voltage-clamp (100 ms pulse in 10 mV steps from -50 to 70 mV) data from WL_2012_ITO. The baseline formulation for *I*_to_ was the same as that presented in the original study. The baseline formulation for *I*_sus_ was taken from Maleckar et al. (2009), derived on human atrial cell data. First, the maximum conductances (*g*_to_, *g*_sus_) were set to give the appropriate magnitude current at the +70 mV clamp step (where each channel is considered to be fully activated). Then, each current was fit to individual, representative experimental current traces [using a least-squares optimization algorithm implemented in Python (Python Software Foundation^2^)] to provide steady-state and time constant values for the activation and inactivation gates at each voltage step, optimized for the numerical solution of the governing equations. The voltage dependence functions describing the steady-states and time constants were then fit to a combination of these values (from a single experiment) and the mean measured values from all experiments [**Figures 1B(i–iii)**], producing current traces from a combined *I*_to_–*I*_sus_ voltage-clamp which match well to experiment [**Figures 1B(iv,v)**].

The conductance parameters were adjusted to reflect the data described in WL_2006 (Workman et al., 2006): setting *g*_to_, the maximal conductance of *I*_to_, to 0.103 nS/pF gave a peak current of 12.5 pA/pF at +60 mV; the proportionally set maximal conductance of 0.040 nS/pF for *I*_sus_ gave a peak current of 5.17 pA/pF, below the value of 10.6 ± 0.8 pA/F described in the data. Therefore, a corrected *I*_sus_ maximal conductance of 0.068 μS/pF was set to reflect these data [**Figure 1B**(**v**-inset)].

#### Parameterizing the Fast Sodium Current, *I*_Na_

**FIGURE 2:**
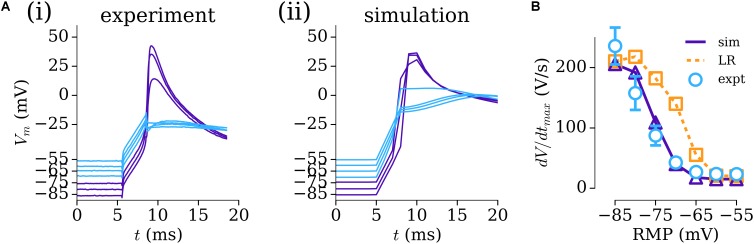
Parameterizing *I*_Na_ through resting membrane potential (RMP) vs dV/dt_max_. **(A)** AP upstroke associated with multiple RMP conditions in experiment **(i)** and simulation **(ii)**. **(B)** Relationship between *dV*/*dt*_max_ and RMP in experiment (open circles, blue; *n* = 10 cells, 4 patients), parameterized *I*_Na_ model (solid-purple line, triangles), and original *I*_Na_ model (Luo and Rudy, 1991) (dotted orange line, squares). Experimental data: unpublished, Priyanka Saxena.

To overcome the challenge of characterizing the voltage-dependence of *I*_Na_, previously unpublished human atrial experimental data (from Priyanka Saxena in the WL) relating the RMP to *dV*/*dt*_max_ were used to modify the parameters of the formulation for *I*_Na_ originally presented by Luo and Rudy, 1991 and used as the baseline in multiple contemporary AP models including those of the human atria (Courtemanche et al., 1998; Grandi et al., 2011). These experimental data were obtained using the same patch-clamp amplifier (chosen for its accurate voltage-following) and recording solutions (including accurate liquid–liquid junction potential correction) as in WL_2012_ITO (Workman et al., 2012) and *dV*/*dt*_max_ measured using WinWCP software (John Dempster). Re-parameterization of this formulation to the data resulted in good agreement between experiment and simulation in the AP upstroke [**Figures 2A(i,ii)**] and dependence of *dV*/*dt*_max_ on RMP (**Figure 2B**), which was significantly different to that observed with the original, ventricular formulation (**Figure 2B**).

#### Workman-Laboratory Characterized Currents: *I*_CaL_

The L-type calcium current, *I*_CaL_, presents a larger challenge to model than the previous currents described due to its tight coupling with the intracellular calcium handling system: the current is responsible for initiating intracellular calcium-induced-calcium-release (CICR) and also has implicit calcium-induced inactivation gating. Detailed electrophysiological data for the human-atrial calcium handling system, and calcium-induced inactivation of *I*_CaL_, are limited (with notable exceptions, e.g., Voigt et al., 2014), and there is limited CaT data available from the WL. Therefore, the formulation for *I*_CaL_ was considered in conjunction with the computational models of the calcium-handling system implemented. Each of the three selected models (hAM_CRN; hAM_NG; hAM_GB; see sections “Materials and Methods” and “Computational Models”) contain different descriptions of CICR and calcium-induced inactivation of *I*_CaL_, and model stability and homeostasis can be sensitive to these components.

**FIGURE 3:**
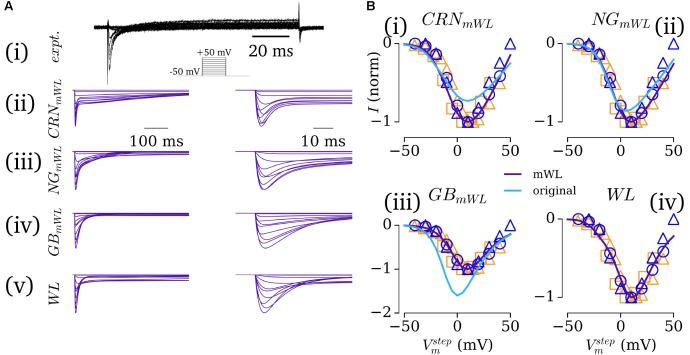
Fitting the L-type calcium current. **(A)** Current traces for experimental (Pau et al., 2007) **(i)** and simulated **(ii–v)** *I*_CaL_ under voltage clamp conditions for the modified WL formulations **(ii–iv)** and novel WL formulation **(v)**, scaled over 500 ms (left) and 50 ms (right). **(B,i–iii)** Comparison of the IV relationship between the modified (purple) and native (light blue) currents against normalized experimental data [dark blue – WL data: circles – WL_2001 (Workman et al., 2001); triangles – WL_2007_ICaL (Pau et al., 2007); orange – data from other labs: Van Wagoner et al. (1999) – squares; Christ et al. (2004) – triangles]. All experimental data are normalized; native simulation data are normalized to the modified peak current, to illustrate the difference in current magnitude between native and modified models. **(B,iv)** The IV relationship for the novel, WL formulation. Figure created using data from: Van Wagoner et al. (1999), Workman et al. (2001), Christ et al. (2004) and Pau et al. (2007).

In the first instance, the native formulations of *I*_CaL_ in each of these models was retained, with only the voltage dependence being modified to fit the experimental IV relationship presented in WL_2007_ICAL and WL_2001 (**Figure 3**), and the maximum conductance set to give a peak current of -5 pA/pF, as reported in WL_2006. Models implementing these formulations of *I*_CaL_ are referred to as the modified-WL models (see section “Computational Models”).

In the second instance, a new formulation for *I*_CaL_ was developed which more closely reproduces the time-course of the experimentally measured current [**Figure 3A(i)**] and was suitable for implementation with both the hAM_CRN and hAM_GB calcium handling systems. The hAM_NG calcium handling system was not used for this purpose due to its sensitivity to changes in *I*_CaL_ and unphysiological CaT magnitude and morphology. The novel formulation implemented the dynamic conductance of *I*_CaL_ of hAM_GB, and was designed to work with both the differing calcium-induced inactivation formulations and CaT time-course associated with each model. This was achieved through first fitting the current formulations (comprising voltage-induced activation and inactivation only) to the voltage clamp data of WL_2007_ICAL, using the same approach as *I*_to_/*I*_sus_ (see section “Workman-Laboratory Characterized Currents: *I*to and *I*sus”). Then, the equations were modified to account for calcium-induced inactivation, with priority on maintaining uniformity in behavior between implementation with each calcium-handling system [**Figures 3A(v),B(iv)**]. This was the formulation used for the full WL models (see section “Computational Models”).

### Computational Models

A family of human atrial cell models is presented which incorporate the formulations for the ion currents described above. First, a minimal approach was used to develop novel models of the isolated-cell human atrial AP: the novel formulations for *I*_Na_, *I*_to_, *I*_sus_, *I*_CaL_, *I*_K1_^isolated^, and *I*_hyp_ were integrated with the intracellular calcium handling models associated with hAM_CRN and hAM_GB (see section “Workman-Laboratory Characterized Currents: *I*_CaL_”); the additional membrane current components necessary for intracellular homeostasis (and unquestionably present in human atrial myocytes; *I*_NaCa_, *I*_NaK_, *I*_CaP_, *I*_Cab_, *I*_Nab_) were introduced using the formulations associated with each calcium handling system. Implementation with the hAM_GB model also retained additional background currents (*I*_Kb_, *I*_Clb_) and other components (*I*_ClCa_). These models, fit as completely as possible to the experimental observations of the WL, are referred to as hAM_WL_CRN_^isolated^ and hAM_WL_GB_^isolated^. Intact-cell variants of these models (hAM_WL_CRN_^intact^, hAM_WL_GB_^intact^) were derived by removal of *I*_hyp_, replacing *I*_K1_^isolated^ with *I*_K1_^intact^, and then minimally introducing *I*_Kr_ and *I*_Ks_ as necessary (see section “Intact-Cell Model”) to maintain physiological human atrial AP duration.

Second, the selected human atrial cell models were modified to reproduce the isolated-cell experimental environments and incorporate the novel ion current formulations: isolated-cell modifications (hAM_X^isolated^) were implemented by the introduction of the new formulation for *I*_K1_^isolated^ and *I*_hyp_, and setting the conductances of *I*_Kr_ and *I*_Ks_ to zero. Modified cell models (hAM_X_mWL_), for both isolated and intact-cells, were implemented by replacing the native formulations of *I*_Na_, *I*_to_, *I*_sus_, and *I*_K1_ with the novel formulations presented, and the formulation of *I*_CaL_ with the modified variants associated with each calcium handling system (see section “Workman-Laboratory Characterized Currents: *I*CaL”). Thus, the present study considers the following models: the three hAM_CRN, hAM_NG and hAM_GB as originally presented; the two novel presented WL based models (hAM_WL_CRN/GB_); and the three modified WL models (hAM_CRN/NG/GB_mWL_); all with isolated an intact variants.

Action potentials were initiated using a train of applied stimuli of magnitude -13.5 pA/pF for 3 ms. All models were paced to stable-state before results were obtained, with a minimum of 200 s pre-pacing stimuli.

## Results

### Isolated-Cell Model Characteristics and Validation

**FIGURE 4:**
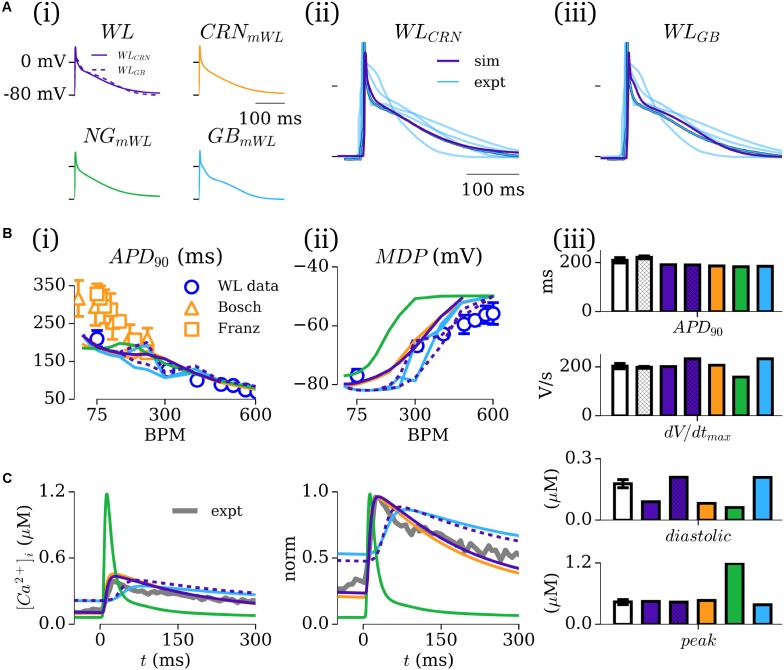
Simulated AP and CaT characteristics and validation. **(A,i)** Action potential (AP) traces associated with the five novel model variants, paced at 75 BPM; **(A,ii,iii)** Comparison between the WL models (purple) and available experimental traces (blue; Workman et al., 2001, 2006, 2012; Pau et al., 2007 + Kettlewell unpublished Ca^2+^ data), with the “typical” Workman laboratory trace emphasized. **(B,i)** APD_90_ restitution for the five models [colored lines – colors correspond to those in A(i)] compared to experimental data from the Workman laboratory (blue circles; WL_2001; Workman et al., 2001) as well as from other groups for additional context [orange triangles (Bosch et al., 1999); orange squares (Franz et al., 1997)]. **(B,ii)** Rate dependence of MDP in the five models and Workman laboratory data (WL_2001). **(B,iii)** Comparison of APD_90_ and *dv*/*dt*_max_ at 75 BPM in the five models (colors; purple hatched bars corresponds to WL_GB_) and experiment (white: WL_2001, open bars; WL_2006, hatched bars). **(C)** Intracellular calcium transients in the five model variants (colored lines) and single experimental trace from the Workman laboratory (black; unpublished Sarah Kettlewell WL data), shown on absolute **(i)** and normalized **(ii)** scales. **(iii)** Comparison of the diastolic and peak calcium in the five models with mean data (*n* = 24 cells, 5 patients) from the Workman laboratory Kettlewell experiments. Figure created using data from Franz et al. (1997), Bosch et al. (1999), Workman et al. (2001, 2006, 2012) and Pau et al. (2007).

The time-courses of the AP for the five novel model variants all exhibit very similar morphologies [**Figure 4A(i)**]; the differences in morphology observed are primarily a result of either the different *I*_CaL_ formulations (see section “Workman-Laboratory Characterized Currents: *I*CaL”), calcium-induced inactivation dependent on the CaT morphology, or the interaction with calcium-handling currents such as *I*_NCX_. Comparison between the model and experimental APs shows good agreement in duration and morphology with the typical WL AP, exhibiting the low and triangular-like plateau morphology [**Figures 4A(ii,iii)**]. APD restitution also matches well with experimental data from WL_2001 [**Figure 4B(i)**], which is in general shorter than the data presented by other groups (Franz et al., 1997; Bosch et al., 1999). The different model variants exhibited different rate dependence of the MDP, with all but the hAM_NG_mWL_ model closely matching the experimental data [**Figure 4B(ii)**]. Comparison of APD_90_ and *dv*/*dt*_max_ between model and experiment (WL_2001 and WL_2006) at 75 BPM showed all of the models exhibit properties close to the experimental value, with variation in *dv*/*dt*_max_ between the models being larger than that for the APD_90_. This is a result of the different RMPs maintained by the consistent hyperpolarizing current.

The intracellular CaT exhibited large differences between model variants dependent on the underlying calcium handling system [**Figure 4C(i)**]: the hAM_NG_mWL_ model retained the very rapid and large amplitude spike associated with the NG model; the CRN and GB variants (WL and modified) exhibited more prolonged CaTs within the physiologically expected amplitude range (∼0.4–0.5 μM). The CRN model variants most closely reproduced the time-course of the CaT [**Figure 4C(ii)**]; the GB model variants exhibited a similar morphology but with a delayed onset. The GB model variants reproduced the diastolic and peak values most closely compared to mean experimental data [**Figure 4C(iii)**].

### Interventions

**FIGURE 5:**
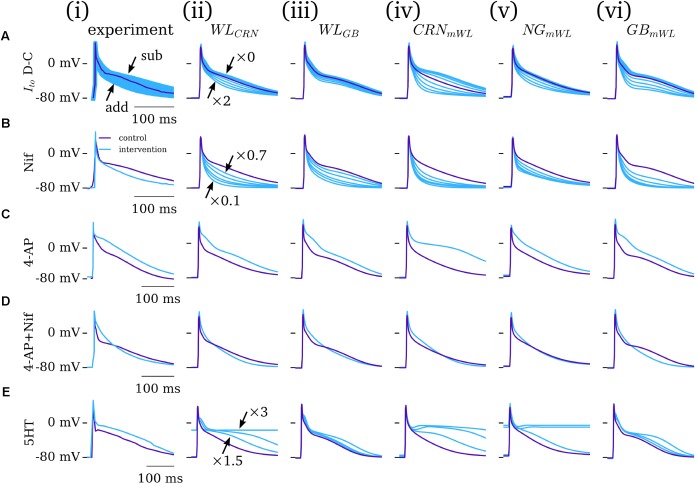
Dynamic clamp and pharmacological intervention. Comparison of experiment **(i)** and five model variants **(ii–vi)** under the application of: **(A)** *I*_to_ dynamic-clamp, modeled as a zero–twofold scaling of the current; **(B)** nifedipine, modeled as a 30–90% block of *I*_CaL_ (specifically 30, 50, 70, 80, and 90% block); **(C)** 4-AP (*I*_to_ and *I*_sus_ block); **(D)** 4-AP + nifedipine; **(E)** 5HT, 1.5 to three-fold scaling of *I*_CaL_. *I*_to_ data from WL_2012_ITO (Workman et al., 2012); Nif and 4-AP data from WL_2001 (Workman et al., 2001); 5HT data from WL_2007_ICAL (Pau et al., 2007). Figure created using data from: Workman et al. (2001, 2012) and Pau et al. (2007).

The five novel model variants (novel WL with CRN/GB calcium handling; CRN/NG/GB WL-modified models) were compared against experimental data from the WL under the application of various pharmacological or dynamic-clamp intervention. The role of *I*_to_ was assessed through comparison to the dynamic-clamp data of WL_2012_ITO [**Figure 5A(i)**]: the magnitude of *I*_to_ was scaled by a factor of zero to two, reproducing the subtraction and addition of up to the full magnitude of current in experiment. All novel model variants reproduced the main features of the intervention, most pertinently the negative correlation between *I*_to_ magnitude and terminal repolarization duration (**Figure 5A**). However, the magnitude of the impact of *I*_to_ modulation was different among the different model variants. As all models contain the same formulation for *I*_to_, these differences were due to either (a) the impact of AP morphology and/or (b) the differing formulations for *I*_CaL_, calcium handling system, and background currents.

The role of *I*_CaL_ was assessed through the application of nifedipine (channel blocker, WL_2001 data, modeled by 30–90% current block) and 5HT (channel enhancer, WL_2007_ICaL data, modeled by a three-fold increase in the current magnitude). The impact of nifedipine was in general overestimated in the cell models [**Figure 5B** – except for than the hAM_NG_mWL_ variant; **Figure 5B(v)**]; 5HT displayed more variable match between model and experiment, with some models overestimating its effects whereas others underestimated it (**Figure 5E**). After-depolarizations or non-repolarizing APs were observed in the hAM_WL_CRN_ and hAM_NG_mWL_ model variants under a large increase in *I*_CaL_. Qualitatively, all models reproduced the features of these two interventions: nifedipine reducing the AP dome/plateau phase and accelerating terminal repolarization, whereas 5HT enhanced this plateau phase and extended terminal repolarization.

The impact of 4-AP (50% block of *I*_t_*_o_* and *I*_sus_) was well reproduced by all model variants, displaying an elevation of the AP plateau and extension of terminal repolarization (**Figure 4C**). Similarly, the match between simulation and experiment for the application of 4-AP combined with nifedipine was strong, wherein all models reproduce the triangular morphology, prolonged phase-1 repolarization, and accelerated terminal repolarization (**Figure 5D**). The extent of APD shortening differed between the model variants, although all displayed some but not extensive shortening, as observed in experiment.

### Intact-Cell Model

**FIGURE 6:**
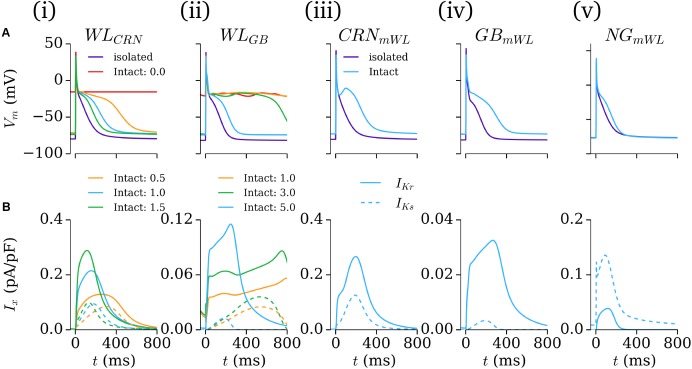
Intact-cell models. **(A)** AP and **(B)** *I*_Kr_ and *I*_Ks_ current traces for the five model variants **(i–v)** under isolated and intact conditions. For panels **(i,ii)**, colors correspond to the isolated-cell model (purple), intact-cell model without introduction of *I*_Kr_ and *I*_Ks_ (red), and introduction of a small amount of each current (orange, green, blue). The scale factors for the WL_CRN_ and WL_GB_ models are indicated in their respective panels. In **iii–v**, just the isolated (purple) and intact (blue) variants are shown. In **B**, solid lines refer to *I*_Kr_ and dotted lines to *I*_Ks_.

The intact-cell models (for future tissue-scale simulations) were derived through first exchanging the formulation of *I*_K1_ from the isolated to intact version (see **Figure 1** and section “Workman-Laboratory Characterized Currents: *I*K1 and *I*hyp”), and subsequent removal of the hyperpolarizing current. The RMP maintained in the cell model variants was ∼-74 mV, similar to that of the native hAM_GB and hAM_NG models, and less hyperpolarized than the isolated-cell model variants, the hAM_CRN model, and WL experiments of ∼-80 mV. For the case of the modified cell models (hAM_X_mWL_), *I*_Kr_ and *I*_Ks_ were modeled as originally presented to preserve the modified nature of the cell models; in case of the WL models (hAM_WL_CRN/GB_), the currents were incrementally introduced by scaling the maximum conductance of the associated model current formulation to preserve the minimal approach: factor of × 0–1.5 for the hAM_WL_CRN_ model, and × 0–5.0 in the hAM_WL_GB_ model. With the conductances set to zero, neither model maintained an APD expected of the intact healthy human atria, exhibiting non-repolarizing APs [**Figures 6A,B(i,ii)**]. In the hAM_WL_CRN_ model, introducing the currents with the conductance set to that presented originally was sufficient to maintain and APD of <400 ms [**Figures 6A,B(i)**]; in the hAM_WL_GB_ model, larger conductances (relative to the original study – note that the GB model has much smaller *I*_Kr_/*I*_Ks_ than the CRN model) were necessary to maintain APD [**Figures 6A,B(ii)**]. All intact models with a duration of <400 ms displayed either a spike-and-dome (hAM_CRN_mWL_) or low-plateau/dome (all other variants) morphology [**Figures 6A,B(i–v)**], and such durations could be achieved with relatively low current magnitudes (i.e., an order of magnitude smaller than the peak in *I*_to_, *I*_sus_, and *I*_CaL_).

### Role of the Hyperpolarizing Current

**FIGURE 7:**
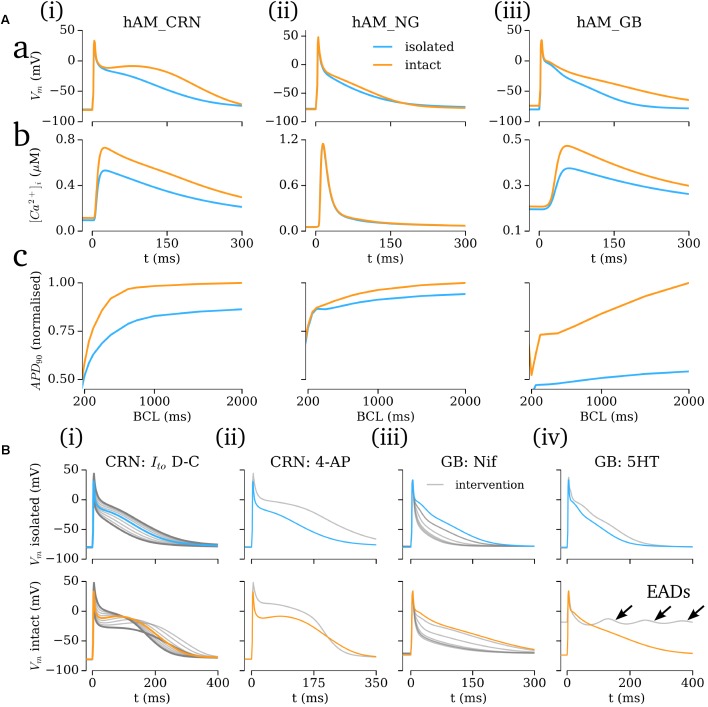
Isolated- vs intact-cell properties. **(A)** Action potentials **(a)**, calcium transient **(b)**, and APD restitution **(c)** plotted against basic cycle length (BCL), for the three baseline cell model **(i–iii)** variants for isolated (blue) and intact (orange). The APD curves were normalized to the intact-cell APD at a cycle length of 2000 ms. **(B)** Comparison of isolated (upper) and intact (lower) cell model response to various interventions (gray). Arrows in **(iv)** lower panel indicate early-after-depolarizations (EADs).

The importance of the hyperpolarizing current, and isolated-cell conditions (i.e., *I*_K1_^isolated^ with a reversal of ∼-40 mV; insignificant *I*_Kr_/*I*_Ks_) was assessed by comparison of the intact -and isolated-cell variants of the hAM_CRN, hAM_NG and hAM_GB models (**Figure 7**). For all three models, the isolated-cell variant exhibited a shorter APD_90_ with more rapid terminal repolarization (**Figure 7Aa**). The dome associated with the hAM_CRN model was flattened, and the long tail associated with the hAM_GB model was significantly reduced. The CaT was significantly reduced in isolated-cells in both the hAM_CRN and hAM_GB models, whereas the hAM_NG model, in general, showed the most similarity between isolated and intact [**Figure 7A(ii)**]. APD restitution was affected similarly, with APD_90_ being significantly shortened at all except the fastest pacing rates (shortest cycle lengths), where convergence between isolated and intact-cell models in the hAM_CRN and hAM_NG model was observed (**Figure 7Ac**).

Analysis of the response of the AP to various interventions revealed some significant differences between isolated- and intact-cell model variants, pertaining both to proportional and behavioral differences. Selected examples illustrate the most significant differences observed (**Figure 7B**): the dynamic clamp *I*_to_ intervention in the hAM_CRN^isolated^ model matched well to the experimental data (**Figure 5A**), wherein terminal repolarization duration negatively correlated with *I*_to_ magnitude, whereas in hAM_CRN^intact^, a positive correlation was observed, wherein initial elevation of the plateau under current block led to more rapid and thus shorter terminal repolarization [**Figure 7B(i)**]. The response to 4-AP in the hAM_CRN model variants demonstrated the same feature: APD_90_ was prolonged in the isolated-cell model but shortened in the intact-cell model [**Figure 7B(ii)**]. In the hAM_GB model variants, modulation of *I*_CaL_ exhibited the largest differences between isolated- and intact-cell models: the application of nifedipine (i.e., *I*_CaL_ current block) exhibited differences in the graded response to gradually increasing current block [**Figure 7B(iii)**]; the application of 5HT (i.e., *I*_CaL_ current enhancement) resulted in early-after-depolarizations (EADs) in the intact-cell model which were not observed in the isolated-cell model [**Figure 7B(iv)**].

### Comparison Between Models

**FIGURE 8:**
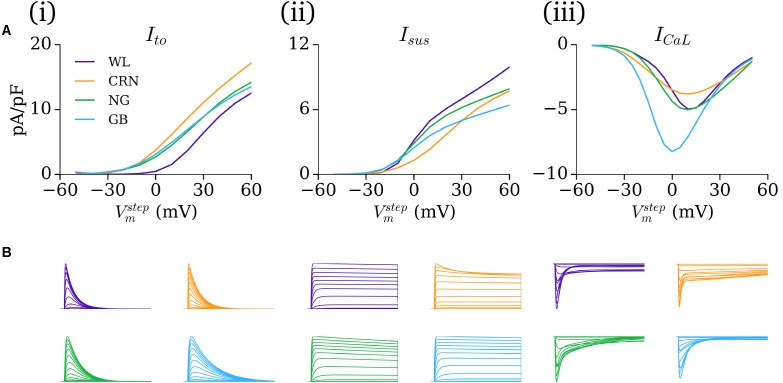
Comparison of currents under voltage clamp. **(A)** Current–voltage (IV) curves and **(B)** Current traces associated with voltage clamp protocols for *I*_to_ **(i)**, *I*_sus_ **(ii)**, and *I*_CaL_ **(iii)** comparing the WL formulations (purple) with those originally presented in the CRN (orange), NG (green), and GB (blue) studies. All current traces are normalized, and presented on the same temporal scale for comparison.

The IV relationship and current traces under voltage clamp for the novel formulations and inherited cell models demonstrate the variability between the different cell models (**Figure 8**). The IV curves for *I*_to_ in the CRN, NG, and GB models all demonstrate a takeoff potential of -30 to -20 mV, whereas the WL formulation exhibits a takeoff potential of -10 to 0 mV, and a smaller current magnitude [**Figure 8A(i)**]. The GB model (i.e., Maleckar et al., 2009 formulation) had the slowest decay time, with all other models being more similar [**Figure 8B(i)**]. *I*_sus_ is largest in the WL model, but exhibits similar voltage dependence to the NG and GB models [**Figure 8A(ii)**]. The model *I*_CaL_ formulations show similar voltage dependence and magnitude for the WL, CRN, and NG models, with the GB model being both larger and with a negative shift in its voltage dependence [**Figure 8A(iii)**]. The inter-model differences in the time-course of *I*_CaL_ traces during voltage clamp are larger, with the WL model showing the fastest decay.

**FIGURE 9:**
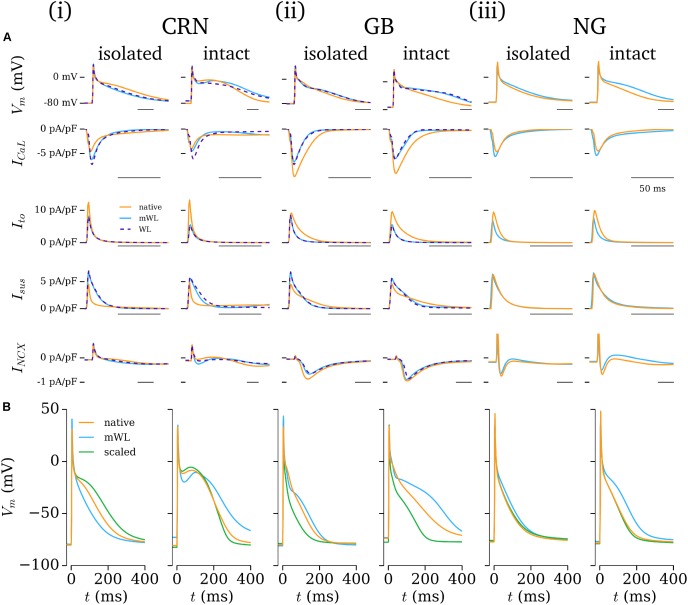
Comparison of excitation and current properties between the different models. **(A)** Comparison of the AP and primary current time-courses associated with the baseline (orange) and modified (blue) cell models, with the full WL model implemented in the baseline model’s calcium handling system shown where relevant (purple dotted). For each baseline model (**i–iii**; CRN, GB, NG), data are shown for both isolated and intact-cell variants (left and right of each panel). The scale bar indicates 50 ms in each panel. **(B)** Comparison of native and modified cell models (orange and blue) with the native model rescaled by the Workman laboratory data (green) for the same cell models and conditions as panel **A**.

The influence of the novel current formulations on atrial AP morphology is illustrated through comparisons of the native (hAM_X), modified (hAM_X_mWL_), and novel (hAM_WL_X_) cell models (**Figure 9A**). In both isolated and intact-cell environments, the AP morphology and time-course of the various currents exhibits significant differences between the native and novel versions, with the isolated modified versions in general exhibiting closer features to the WL AP morphology and duration. The time-courses for the novel *I*_to_ and *I*_sus_ lie somewhat intermediary to those associated with the hAM_CRN and hAM_GB models.

The contribution of ion current balance (i.e., relative magnitudes of the different currents) and kinetics to the differences observed was assessed by comparing the native and mWL models with variants in which the current magnitudes of the native models were scaled to the same data as the WL models (WL_2006). These data highlight that both current magnitude and specific kinetics contribute to the differences observed in the mWL models, as the rescaled model variant did not reproduce the morphology of the mWL model (**Figure 9B**).

## Discussion

### Summary

In this study, mathematical descriptions of the primary ion currents underlying human atrial electrophysiology were developed based primarily on congruent human atrial cell data from a single source, and specifically reproducing the environment of isolated atrial cardiomyocyte whole-cell-patch current- and voltage-clamp experiments, including the frequently observed unphysiologically depolarized RMP and injected compensating hyperpolarizing current. Formulations describing the currents *I*_to_, *I*_sus_, *I*_CaL_, and *I*_K1_ were all fit to voltage-clamp data from the WL (**Figures 1**, **3**); *I*_Na_ was parameterized using experiments relating RMP to *dV*/*dt*_max_ (**Figure 2**). The formulations were integrated with multiple available existing models of human atrial electrophysiology which include descriptions of intracellular calcium handling, to produce a family of novel and modified models of the human atrial myocyte (**Figure 4**). The models were compared to experimental data describing AP morphology, APD, rate-adaption, and current magnitudes (**Figure 4**) as well as pharmacological and dynamic clamp interventions (**Figure 5**); model variants were also compared to each other to elucidate the role of the primary ion current parameters in human atrial cell dynamics (**Figures 8**, **9**), and the important differences between isolated- and intact-cell environments were characterized (**Figure 7**).

### Comparison to Previous Work

There are many available models describing human atrial electrophysiology developed by multiple groups. Comprehensive reviews and comparisons of these models can be found in Wilhelms et al. (2013), Trayanova (2014), and Heijman et al. (2016), but these models can be briefly categorized into a few independent models (Courtemanche et al., 1998; Nygren et al., 1998; Grandi et al., 2011) and variants of these, introducing, for example, reformulations due to new data (Maleckar et al., 2009), mutant ion channel variants (Colman et al., 2017a), parameterization to patient specific data (Lombardo et al., 2016), or population variability models (Ellinwood et al., 2017; Lawson et al., 2018; Ni et al., 2018). Whereas these models are typically based on data from multiple sources, cell-types, or species, the ion current formulations developed in the present study are derived directly from human atrial specific data from a single laboratory. Importantly, where many previous studies have adjusted current parameters to reproduce AP properties, no such modifications were made in the present study, ensuring that the relative balance and time-courses of the primary currents is experimentally justified. Furthermore, the model presented is the first to explicitly implement the conditions of isolated-cell experiments and to specifically differentiate isolated- and intact-cells. Analysis presented in this study highlights the importance of reproducing these isolated-cell environmental conditions when considering validation and comparison of AP response to intervention (sections “Role of the Hyperpolarizing Current” and “The Importance of the Isolated-Cell Environment”). Therefore, the models presented offer advantages for general integrated experimental-numerical investigation compared to previously presented models which do not reproduce these conditions, as well as for direct integration with experiments in the WL for which the model is tailored. Similar approaches could be applied to develop specific models for other laboratories and experimental environments; incidentally, a comparison of such models may provide the best means to fully assess the impact of inter-laboratory differences and elucidate the role and variation of ion currents in human atrial electrophysiology.

The single-source approach implemented theoretically provides a higher level of confidence than previous models, due to the absence of arbitrary or results-based parameter modification and lack of ambiguity in combination of the formulations of the primary ion currents. However, this is far from a claim that it is therefore superior, or that the previous models are “less-valid”; rather, at the very least, the formulations presented in this study, and the models which implement them, exhibit differences to those previous models (section “Comparison Between Models”) and therefore represent complementary tools which may assist in multi-model studies to provide a further, independent perspective and thus achieve model-independent conclusions.

### The Importance of the Isolated-Cell Environment

Isolated human atrial cardiomyocytes frequently feature unphysiologically depolarized RMP (Escande et al., 1987; Bénardeau et al., 1996; Dobrev et al., 2001; Workman et al., 2006) and apparent reversal of *I*_K1_ at ∼40 mV. This issue has been addressed in a variety of ways; e.g., by current-clamping with a small, constant, hyperpolarizing current (Le Grand et al., 1994; Bénardeau et al., 1996; Workman et al., 2001). However, the previous models do not explicitly account for such RMP depolarization or hyperpolarizing current application (although studies in ventricular cells have investigated the effect of applied constant currents on APD at different rates, e.g., Grandi et al., 2010). Results presented in this study (section “Role of the Hyperpolarizing Current”), and based on the available, well-established human atrial cell models (i.e., independent of novel ion current formulations), demonstrated that these features can lead to significantly different behavior compared to “intact”-cells (i.e., the models as natively presented, with *I*_K1_ reversal ∼*E*_K_ and a well maintained RMP of more negative than -70 mV): all isolated-cell model variants exhibited a shorter APD_90_ and a smaller CaT than the intact equivalents. In the hAM_CRN model, the different environments resulted in the difference between “spike-and-dome” versus “low-dome/triangulated” AP morphologies, which may have a significant impact on model dynamics.

Furthermore, the models responded differently to the application of interventions, in some cases in significant ways such as APD shortening vs lengthening, or the emergence of EADs. Some of these differences may simply be a result of AP morphology and its influence on the time-course of the underlying ion currents, but it is also highly likely that the more positive reversal potential and constant, injected hyperpolarizing current, as well as the absence of *I*_Kr_/*I*_Ks_, more directly underlies these differences to at least some extent (*I*_Kr_ and *I*_Ks_, for example, being responsible for accelerated terminal repolarization associated with AP plateau elevation in intact-cell models).

These results therefore highlight the considerations which must be taken when validating human atrial cell models against experimental data attained under these isolated-cell conditions, for translation of phenomena observed under perturbed conditions to the tissue scale, and for experimentally assessing model predictions attained without accounting for these conditions.

### Potential Contributions of *I*_Kr_ and *I*_Ks_ to Human Atrial Repolarization

Due to *I*_Ks_ and *I*_Kr_ being very small or absent (Firek and Giles, 1995; Amos et al., 1996; Schreieck et al., 2000; Caballero et al., 2010) in isolated human atrial cells, these currents were not included in the isolated-cell model. Repolarization kinetics and duration properties matched well with experimental traces, indicating that these currents are not required for repolarization under these conditions. Extrapolation to the intact-cell model, in which *I*_hyp_ was removed, indicated that *I*_Kr_/*I*_Ks_ may be required in the intact human atria to maintain repolarization, as cell models exhibited prolonged (>700 ms) or non-repolarizing APs without inclusion of these currents. However, inclusion of these currents to only a relatively small magnitude was sufficient to maintain AP repolarization.

### Limitations

Whereas the primary ion currents controlling the AP model were fit to detailed data from a single laboratory, the intracellular Ca^2+^ handling system was not parameterized to the same extent to human atrial data, and only limited CaT data were available for validation from the WL. For this reason, the current formulations were integrated with multiple contemporary human atrial calcium handling systems, rather than performing development and parameterization of a novel calcium handling system. However, the development of a more rigorously derived human atrial specific calcium handling system (for example, an extension of the spatial cell model presented in Voigt et al., 2014) would be a natural improvement to a full human atrial single-source model. Furthermore, integration with multiple, independent systems provided challenges for modeling the L-type calcium current homogeneously across the different models, due to the tight and sensitive coupling between this current and the calcium handling system. This led to a degree of uncertainty and subjectivity in the selection of parameters from non-unique solutions, which contrasts with the single-source and objective philosophy of the approach.

In general, the models and variants overestimated the impact of high concentrations of nifedipine on human atrial AP shortening. Whereas this feature is common to many models of the human atria, and in particular in the isolated-cell variants as presented in this study, its emergence in a model containing accurately reproduced dynamics for the main ion currents characterized in experiments suggests a potential important role of inward currents active at potentials more negative than -30 mV not captured in the model, possibly because *I*_NCX_ is too small, or the presence of larger background Ca^2+^ and Na^2+^ currents. This type of insight, however, supports a primary purpose of the model: for example, the potential role of incorrect relative current magnitude balance in the formulations of the primary currents underlying this discrepancy can be largely eliminated.

Variability was not investigated in the present study, although the single-source approach may offer the perfect tool to interpret data attained from the implementation of a full population variability approach and/or quantification through sensitivity analysis (Chang et al., 2015; Devenyi et al., 2017; Ellinwood et al., 2017; Sánchez et al., 2017; Lawson et al., 2018; Ni et al., 2018).

Due to the single-source, minimalistic philosophy of the approach, there are many factors which play a role in human atrial electrophysiology which have not been included, due to a lack of well characterized and human atrial specific data. For example, the models do not contain detailed descriptions of phosphorylation due to PKA, or the impact of CaMKII (and serine and threonine; Heijman et al., 2014, 2017; Mesubi and Anderson, 2016), or additional currents such as small conductance potassium channels (Skibsbye et al., 2014), which may have an important influence on human atrial cell dynamics in control and perturbed conditions. Exclusion of these components is not a claim that they are unimportant, but rather follows from the motivation not to introduce sophistication and complexity by including components which have not been well characterized in the human atria (and specifically the WL) and thus contain potential sources of error, *ad hoc* parameters, or inaccurately scaled contributions. We hope that in future further, well-characterized components will be added to the model as part of the long-term ambition of the biophysically detailed and biomedically accurate virtual human heart.

## Conclusion

We have developed a family of biophysically detailed models of human atrial electrophysiology based primarily on data from a single laboratory and reproducing the conditions of isolated-cell experiments. The models reproduce human atrial excitation properties and the impact of various interventions, and the importance of the isolated-cell environment was highlighted. We therefore present a tool which can be used directly in conjunction with experiments to dissect the ionic mechanisms of atrial arrhythmias, and complementary to the available contemporary cell models for general mechanistic investigation.

## Author Contributions

MC and AW conceived and designed the study. MC carried out simulations, and acquired and analyzed the data. PS, SK, and AW performed isolated-cell experiments, and acquired and analyzed the data. MC prepared all the figures. MC prepared and all authors edited the drafts and manuscript. All authors approved the final version of the manuscript, interpreted the data, and agreed to be accountable for all aspects of the work in ensuring that questions related to the accuracy or integrity of any part of the work are appropriately investigated and resolved.

## Conflict of Interest Statement

The authors declare that the research was conducted in the absence of any commercial or financial relationships that could be construed as a potential conflict of interest.

## Footnotes

1 https://github.com/michaelcolman/hAM_WL

2 https://www.python.org/
