# Supplementary material for: Description of the Human Atrial Action Potential Derived From a Single, Congruent Data Source: Novel Computational Models for Integrated Experimental-Numerical Study of Atrial Arrhythmia Mechanisms
Source: Front Physiol. 2018 Sep 7;9:1211. doi: 10.3389/fphys.2018.01211 (PMC6137999; doi:10.3389/fphys.2018.01211)
Supplement: Supplementary file 1 [file Data_Sheet_1.pdf]

Supplementary Material

Description of the human atrial action potential derived from a single, congruent data source: Novel computational models for integrated experimental-numerical study of atrial arrhythmia mechanisms

Michael A. Colman*, Priyanka Saxena, Sarah Kettlewell, Antony J. Workman

*** Correspondence:** m.a.colman@leeds.ac.uk

Contents

1 Novel current formulations 2
1.1 Time independent potassium current, I_{K1} 2
1.2 Rapid potassium currents, I_{to} and I_{sus} 2
1.3 L-type calcium current, I_{CaL} 3
1.4 Fast-sodium current, I_{Na} 3
2 Implementation with cell models 5
2.1 The minimal, Workman-lab models 5
2.2 Modified cell models 5
3 Parameters 6
4 References 6

1 Novel current formulations

1.1 Time independent potassium current, I_{K1}

Due to the importance of the form of the current in the voltage range -80mV - -40 mV, where the current may be very small, polynomials were used to achieve a precise fit to the complex voltage dependence of the current:

$$I_{K1}^{isolated} = 4(0.096 + 7.12\times10^{-3}V_m + 8.95\times10^{-5}V_m{}^2 - 7.13\times10^{-8}V_m{}^3 - 1.35\times10^{-9}V_m{}^4) \quad (1)$$

$$I_{K1}^{intact} = 4(0.0029 + 1.29\times10^{-3}V_m + 3.51\times10^{-5}V_m{}^2 - 9.76\times10^{-8}V_m{}^3 - 1.35\times10^{-9}V_m{}^4) \quad (2)$$

1.2 Rapid potassium currents, I_{to} and I_{sus}

$$I_{to} = g_{to}.va_{Ito}.\left((1-F_S)vi_{Ito\_1} + F_s vi_{Ito\_2}\right).(V_m - E_K) \quad (3)$$

$$I_{sus} = g_{sus}.va_{Isus}.vi_{Isus}.(V_m - E_K) \quad (4)$$

Where the dynamics of the gating variables ($va_{ito/sus}$, $vi_{ito/sus}$) are described by the general differential equation:

$$dx/dt = (x_{ss} - x)/x_\tau \quad (5)$$

With steady-states:

$$va_{Ito\_ss} = 1/\left(1 + e^{-(V_m - 15)/7}\right) \quad (6)$$

$$vi_{Ito\_1\_ss} = 1/\left(1 + e^{(V_m - (-23)/5.3}\right) \quad (7)$$

$$vi_{Ito\_2\_ss} = vi_{Ito\_1\_ss} \quad (8)$$

$$va_{Isus\_ss} = 1/\left(1 + e^{(V_m - (-4.25))/5.61}\right).4.15.e^{0.183V_m - 0.9849} \quad (9)$$

$$vi_{Isus\_ss} = 1/\left(1 + e^{(V_m - (-7.5))/10}\right) \quad (10)$$

And time constants:

$$va_{Ito\_\tau} = 0.4 + 18e^{-\left(\frac{V_m + 40}{45}\right)^2} \quad (11)$$

$$vi_{Ito\_1\_\tau} = 8.6 + 62e^{-\left(\frac{V_m + 32}{27}\right)^2} \quad (12)$$

$$vi_{Ito\_2\_\tau} = 15 + 29.73/\left(1 + e^{0.0696(V_m - 2.72)}\right) \quad (13)$$

$$va_{Isus\_\tau} = 0.5 + 0.9 / \left(1 + e^{(V_m+5)/12}\right) \quad (14)$$

$$vi_{Isus\_\tau} = 3000 + 590 / \left(1 + e^{(V_m+60)/10}\right) \quad (15)$$

And proportion of fast/slow channels given by:

$$F_s = 0.2 / \left(1 + e^{-(V-35)/5}\right) \quad (16)$$

1.3 L-type calcium current, I_{CaL}

The novel formulation presented has the following form:

$$I_{CaL} = p_{CaL}.va_{ICaL}.(0.8vi_{ICaL\_1} + 0.2vi_{ICaL\_2}).ci_{ICaL}.\bar{I}_{CaL,Ca} \quad (17)$$

Voltage-dependent gates:

$$va_{ICaL\_ss} = 1 / \left(1 + e^{-(V_m-0.5))/5.967}\right) \quad (18)$$

$$vi_{ICaL\_1\_ss} = 1 / \left(1 + e^{(V_m-(-18))/3.8}\right) \quad (19)$$

$$vi_{ICaL\_2\_ss} = vi_{ICaL\_1\_ss} \quad (20)$$

$$va_{ICaL\_\tau} = 7.02 - 2.37e^{-((V_m-14.45)/52.33)^2} \quad (21)$$

$$vi_{ICaL\_1\_\tau} = 16.48 - 10.72e^{-((V_m-(-2.22))/22.64)^2} \quad (22)$$

$$vi_{ICaL\_2\_\tau} = 12424 - 12027e^{-((V_m-13)/83)^2} \quad (23)$$

Where $I_{CaL,Ca}$_bar was modelled as presented in Grandi et al. 2011. Calcium inactivation was modelled as in the baseline Ca^{2+}-handling system, with the following modifications for the WL models (not the modified models, which retain calcium-inactivation as originally presented):

$$ci_{ICaL\_\tau} = 50.0 \quad \} CRN \quad (24)$$

$$\left.\begin{aligned} ci_{ICaL\_\alpha} &= 5.1 \\ ci_{ICaL\_\beta} &= 8.33 \times 10^{-3} \end{aligned}\right\} GB \quad (25)$$

1.4 Fast-sodium current, I_{Na}

$$I_{Na} = g_{Na}.va^3_{INa}.vi_{INa\_1}.vi_{INa\_2}.(V_m - E_{Na}) \quad (26)$$

$$va_{INa\_\alpha} = 0.32\left(V_m + 39.13\right) / \left(1 + e^{-0.09(V_m+39.13)}\right) \quad (27)$$

$$va_{INa\_\beta} = 0.08e^{-(V_m-8)/11.0} \quad (28)$$

If V_m < -40 mV:

$$vi_{INa\_1\_\alpha} = 0.135e^{-(V_m+85)/6.8} \tag{29}$$

$$vi_{INa\_1\_\beta} = 3.285e^{0.079(V_m+5)} + 31000e^{0.35(V_m+5)} \tag{30}$$

$$vi_{INa\_2\_\alpha} = \left(-127140e^{0.24444(V_m+5)} - 3.474\times10^{-5}e^{-0.04391(V_m+5)}\right)\left(\frac{V_m+42.78}{1+e^{0.3111(V_m+84.23)}}\right) \tag{31}$$

$$vi_{INa\_2\_\beta} = 0.10908e^{-0.01052(V_m+5)} / \left(1+e^{-0.1378(V_m+45.14)}\right) \tag{32}$$

Else:

$$vi_{INa\_1\_\alpha} = 0 \tag{33}$$

$$vi_{INa\_1\_\beta} = 1/\left(0.13(1+e^{-(V_m+15.86)/11.1})\right) \tag{34}$$

$$vi_{INa\_2\_\alpha} = 0 \tag{35}$$

$$va_{INa\_2\_\beta} = 0.3e^{2.535\times10^{-7}(V_m+5)} / \left(1+e^{-0.1(V_m+37)}\right) \tag{36}$$

And the steady state and time constant for each gate defined by:

$$v_{x\_ss} = v_{x\_\alpha} / \left(v_{x\_\alpha} + v_{x\_\beta}\right) \tag{37}$$

$$v_{x\_\tau} = 1/\left(v_{x\_\alpha} + v_{x\_\beta}\right) \tag{38}$$

2 Implementation with cell models

2.1 The minimal, Workman-lab models

The WL model integrated with the CRN[1] (WL_{CRN}) retained the additional components (I_{NaCa}, I_{NaK}, I_{CaP}, I_{Cab}) and background currents (I_{Nab}) as presented in the original study without modification. Implementation with the Grandi et al. 2011 model [2] (WL_{GB}) includes these components as well as the additional inclusion of I_{Kb}, I_{ClCa}, I_{Clb} from the inherited model. The conductance of these additional currents was reduced in the isolated cell model ($g_{Kb} \times 0.2$, g_{ClCa}, $g_{Clb} \times 0.5$ – isolated cell variants only). Furthermore, integration with the Grandi calcium handling system required modifications to maintain calcium homeostasis: the maximal flux rates for the following parameters were adjusted: J_{leak} and $I_{CaP} \times 0.3$; $I_{NCX} \times 0.64$. This was performed for both the novel and modified cell models, isolated- and intact- environments.

2.2 Modified cell models

The modified cell models for the CRN, Grandi and Nygren et al. [3] were created by replacing I_{Na}, I_{to}, I_{sus}. I_{K1} with the novel formulations, and modifying the steady states of the voltage dependence of I_{CaL} to fit the experimental IV relationship and current magnitude of the WL data. The modifications were as follows:

CRN model: 3 mV positive shift in voltage dependence of all functions; gradient parameter of activation gate changed from 7.45 to 7.07; increase in current conductance of 1.725.

Grandi model: 10 mV positive shift in the voltage dependence of all functions; gradient parameter of activation gate changed from 7.2 to 6.84; decrease in current conductance of 0.9.

Nygren model: 4 mV positive shift in the voltage dependence of all functions; gradient parameter of activation gate changed from 5.8 to 6.44; increase in current conductance of 1.28. To maintain calcium homeostasis, it was also necessary to increase I_{CaP} (×1.5) and I_{Kr}/I_{Ks} (×5) and decrease I_{Cab} (×0.3) in the intact variant.

3 Parameters

Symbol	Parameter	Value
$[K^+]_o$	External potassium concentration (mM)	4
$[Na^+]_o$	External sodium concentration (mM)	140
$[Ca^{2+}]_o$	External calcium concentration (mM)	1.8
g_{Na}	Maximal conductance I_{Na} (nS/pF)	17.55
g_{to}	Maximal conductance I_{to} (nS/pF)	0.1028
g_{sus}	Maximal conductance I_{sus} (nS/pF)	0.0676
p_{CaL}	Maximal flux rate I_{CaL} (cm/s) – hAM_WL$_{CRN}$	5.3 $\times 10^{-4}$
p_{CaL}	Maximal flux rate I_{CaL} (cm/s) – hAM_WL$_{GB}$	6.4 $\times 10^{-4}$

C/C++ code available from https://github.com/michaelcolman/hAM_WL_model and http://physicsoftheheart.com/ .

4 References

1. Courtemanche M, Ramirez RJ, Nattel S. Ionic mechanisms underlying human atrial action potential properties: insights from a mathematical model. Am J Physiol. 1998 Jul;275(1 Pt 2):H301-321.

2. Grandi E, Pandit SV, Voigt N, Workman AJ, Dobrev D, Jalife J, et al. Human Atrial Action Potential and Ca2+ Model: Sinus Rhythm and Chronic Atrial Fibrillation. Circ Res. 2011 Oct 14;109(9):1055–66.

3. Nygren A, Fiset C, Firek L, Clark JW, Lindblad DS, Clark RB, et al. Mathematical Model of an Adult Human Atrial Cell: The Role of K+ Currents in Repolarization. Circ Res. 1998 Jan 23;82(1):63–81.
